# Knockout of *Drosophila* RNase Z^L^ impairs mitochondrial transcript processing, respiration and cell cycle progression

**DOI:** 10.1093/nar/gkv1149

**Published:** 2015-11-08

**Authors:** Xie Xie, Edward B. Dubrovsky

**Affiliations:** 1 Department of Biology, Fordham University, Bronx, NY 10458, USA; 2 Center for Cancer, Genetic Diseases, and Gene Regulation, Fordham University, Bronx, NY 10458, USA

## Abstract

RNase Z^L^ is a highly conserved tRNA 3′-end processing endoribonuclease. Similar to its mammalian counterpart, *Drosophila* RNase Z^L^ (dRNaseZ) has a mitochondria targeting signal (MTS) flanked by two methionines at the N-terminus. Alternative translation initiation yields two protein forms: the long one is mitochondrial, and the short one may localize in the nucleus or cytosol. Here, we have generated a mitochondria specific knockout of the *dRNaseZ* gene. In this *in vivo* model, cells deprived of dRNaseZ activity display impaired mitochondrial polycistronic transcript processing, increased reactive oxygen species (ROS) and a switch to aerobic glycolysis compensating for cellular ATP. Damaged mitochondria impose a cell cycle delay at the G_2_ phase disrupting cell proliferation without affecting cell viability. Antioxidants attenuate genotoxic stress and rescue cell proliferation, implying a critical role for ROS. We suggest that under a low-stress condition, ROS activate tumor suppressor p53, which modulates cell cycle progression and promotes cell survival. Transcriptional profiling of p53 targets confirms upregulation of antioxidant and cycB-Cdk1 inhibitor genes without induction of apoptotic genes. This study implicates *Drosophila* RNase Z^L^ in a novel retrograde signaling pathway initiated by the damage in mitochondria and manifested in a cell cycle delay before the mitotic entry.

## INTRODUCTION

Cells of metazoans contain two populations of tRNA molecules encoded by nuclear and mitochondrial DNA. To become functional, all transfer RNAs undergo maturation process as initially nuclear-encoded tRNAs are transcribed with 5′- and 3′-extentions, and mitochondrial tRNAs are embedded in long polycistronic primary transcripts. Generation of correct tRNA ends involves endonucleolytic cuts by RNase P, either nuclear or mitochondrial 5′-end ribonuclease, and RNase Z^L^, a 3′-end ribonuclease (1,2). While two RNase P enzymes are each targeted to different cellular compartments, the sole RNase Z^L^ is localized in both nucleus and mitochondria.

Mitochondria are double-membrane organelles with their own genome. They are primarily known as the site of ATP synthesis via oxidative phosphorylation (OXPHOS). The mitochondrial respiratory chain is organized in four electron-transporting complexes (I–IV) and a proton translocating complex (V), all made up of approximately 100 polypeptides most of which are encoded in the nucleus. Mitochondrial DNA (mtDNA) is a small circular molecule, multiple copies of which are located in the mitochondrial matrix. It encodes 13 polypeptide subunits of respiratory complexes I, III, IV and V, plus the RNA components of the mitochondrial translational apparatus – 2 ribosomal RNAs (mt-rRNAs) and 22 transfer RNAs (mt-tRNAs). The mtDNA of *Drosophila melanogaster*, consisting of 19 517 bp, is transcribed from both strands generating five polycistronic RNA species (3,4). Processing of the large primary RNAs is aided by the spontaneous folding of the cloverleaf tRNA structures serving as punctuation marks separating individual transcripts (5). Endonucleolytic cuts liberating mature mt-tRNAs also yield functional mitochondrial mRNAs and rRNAs competent for translation and ribosome assembly. Therefore, RNase Z^L^ activity is critical not only for mt-tRNA 3′-end processing, but for overall mitochondrial genome expression. Some studies imply that *RNase Z^L^* mutations may impair mitochondrial respiration (6,7), however, the details of mitochondrial damage and how the impaired bioenergetics is manifested at the cellular level have not been addressed yet.

Mitochondria are not an independent component of eukaryotic cell. Functional mitochondria rely on the import of hundreds of nuclear-encoded proteins (8,9). Furthermore, mitochondria themselves can incite intracellular signaling pathways collectively known as a retrograde regulation that could change cellular physiology (10). Cells constantly monitor mitochondrial functionality and respond to any changes to accommodate themselves to organelle deficiencies. Often, mitochondrial dysfunction is associated with a rise in AMP levels and reactive oxygen species (ROS) production. Both AMP and ROS are active signaling molecules that can elicit retrograde responses and coordinate mitochondrial bioenergetics with cellular proliferation. As mechanisms of the retrograde signaling in metazoans start to emerge, two different pathways both enforcing the G_1_/S checkpoint of cell cycle were identified in *Drosophila* (11). Mutations disrupting complexes I and IV of the electron transport chain (ETC) cause the G_1_/S arrest either through ROS mediated JNK activation or AMP mediated AMPK activation. Pathways targeting retrograde regulation at the G_2_/M checkpoint have not been identified yet. RNase Z^L^ is one of nuclear-encoded proteins targeted to mitochondria. Importantly its knockout delays cell cycle specifically at G_2_/M (12). Finding the mechanism of retrograde regulation in this case has been challenged by multiple duties of RNase Z^L^ in nucleus, cytosol and mitochondrion.

The fly genome contains a sole gene, *dRNaseZ*, that encodes enzyme with the tRNA 3′-end endonucleolytic activity. *Drosophila* RNase Z^L^ (dRNaseZ) has at least two functions – nuclear pre-tRNA processing and mitochondrial primary transcript processing (13,14). Taking advantage of *Drosophila* genetics we generated a fly model, in which intra-mitochondrial dRNaseZ activity is separated from its other functions. We found that loss of mitochondrial dRNaseZ abrogates ETC, increases ROS formation and, overall, results in a low-level genotoxic stress and the G_2_/M delay. Antioxidants reduce ROS levels and relieve the cell cycle arrest. Our results point at a novel retrograde signaling pathway connecting mitochondrial dysfunction and cell cycle progression.

## MATERIALS AND METHODS

### Fly stocks

Flies from the *Drosophila* Stock Center in Bloomington: *FRT^G13^,L/SM6a* (FBst0001958), *FRT^G13^,GFP^nls^* (FBst0005826), *hsFLP^22^* (FBst0008862), and *M(2)l/SM1* (FBst0000342), *L,Pin/CyO,GFP* (FBst0005194). The *FRT^G13^,GFP^nls^,M(2)l* chromosome was generated by recombination. The *dRNaseZ* knockout flies (*RNZ^ED24^*) and transgenic lines carrying *hs-RNZ-V5* (*hsRNZ*) or *genRNZ^WT^-V5* (*genRNZ^WT^*) constructs were established and described previously (12). The *genRNZ^ΔMTS^-V5* construct was generated with the pCa4B2G vector (a gift from Dr Perrimon) using Site-Directed Mutagenesis Kit (Stratagene) and primers genRNZM1LF and genRNZM1LR (Supplementary Table S1). Transgenic line was established with the φC31-mediated transformation into the attP site at 68A. For conditional rescue, control *RNZ^ED24^/CyO,GFP;genRNZ^ΔMTS^*/*hsRNZ* and mutant *RNZ^ED24^;genRNZ^ΔMTS^*/*hsRNZ* larvae were subjected to 1 h heat shock (HS) at 37°C on day 1 after egg deposition (AED).

### Plasmids, cell culture and cell transfection

The open reading frame of dRNaseZ was PCR-amplified on the EST clone template (SD27051, *Drosophila* Genomic Resource Center) with primers containing KpnI (KpnI–RNZ) and XhoI (XhoI–RNZ) restriction sites (Supplementary Table S1), and cloned into the pMT/V5-His vector (Invitrogen). *RNZ^ΔMTS^* was created with the Mutagenesis Kit and primers pMTRNZM1LF and pMTRNZM1LR (Supplementary Table S1). All constructs were verified by sequencing, and correct expression was confirmed by Western blot with anti-V5 antibodies. *Drosophila* S2 cultured cell maintenance and transient transfection was performed as described previously (15). Protein expression was induced with 0.5 mM CuSO_4_.

### Northern and Western blot analysis

Northern and Western blot analyses were performed as previously described (12). Northern probes are listed in the Supplementary Table S2. Primary antibodies used in Western were mouse anti-V5 (Invitrogen, 1:10 000), mouse anti-α-tubulin (Sigma, 1:5000), rabbit anti-γH2Av (Rockland, 1:1000) and rabbit anti-histone H3 (Millipore, 1:25 000). Secondary antibodies from Jackson ImmunoResearch were Peroxidase-conjugated goat anti-mouse (1:10 000) and Peroxidase-conjugated goat anti-rabbit (1:10 000).

### Immunostaining and microscopy

Transfected S2 cells were plated onto concanavalin A coated coverslips, stained with 250 nM MTRed (MitoTracker Red CMXRos, Invitrogen), and fixed with 4% formaldehyde before incubating with mouse anti-V5 (1:200) overnight at 4°C. Secondary antibody FITC-goat anti-mouse (Jackson ImmunoResearch) was used at 1:200. Nuclei were counterstained with 5 μg/ml Hoechst 33258 (Sigma). Cells were mounted in Fluoromount-G and analyzed with confocal microscope, Leica TCS SP5.

Immunostaining of imaginal discs was performed as previously described (12). The following antibodies were used: mouse anti-BrdU (Roche, 1:100), rabbit anti-PH3 (Millipore, 1:500), rabbit anti-γH2Av (Rockland, 1:500) and rabbit anti-cleaved Casp3 (Cell Signaling, 1:200). The secondary antibodies were from Jackson ImmunoResearch: FITC-goat anti-rabbit (1:200), Cy3-goat anti-mouse (1:400) and Cy5-goat anti-rabbit (1:400). Samples were analyzed with a Zeiss Axio Imager M1 microscope.

Immunostaining in spermatids was performed according to White-Cooper (16). For BrdU labeling, eye discs were incubated in 75 μg/ml BrdU for 30 min, then washed with PBS, fixed in 1% formaldehyde and 0.01% tween-20 overnight at 4°C, and treated with 0.05 U/μl DNase I (Roche) for 2 h at 37°C before immunostained as described above. For mitochondrial staining, third-instar imaginal discs were incubated in 100 nM MTRed for 10 min. Images were collected immediately. For ROS detection, DHE staining was adapted from (11). All images were analyzed using AxioVision 4.9, ImageJ and Adobe photoshop CS6.

### Mosaics

Mosaic clones were obtained using the FLP/FRT technique. *hsFLP;FRT,RNZ^ED24^/FRT,GFP;genRNZ* (for clones in a WT background) or *hsFLP;FRT,RNZ^ED24^/FRT,GFP,M;genRNZ* larvae (for clones in a *Minute* background) were HS for 90 min at 37°C on second day AED, and dissected on fifth day AED.

### Antioxidant treatment

After HS treatment, larvae were transferred to food containing 160 μg/ml AD4 (Sigma), 5 mg/ml NAC (Sigma) or water (solvent control).

### Flow cytometry

For 10 WT and 20 *hsRNZ* conditionally rescued *RNZ^ΔMTS^* larvae, flow cytometry analysis of third-instar wing disc cells was performed as previously described (12). For mosaic larvae, flow cytometry analysis of third-instar wing discs with WT or *RNZ^ΔMTS^* clones were adapted from Neufeld *et al*. (17); cells were analyzed with a BD LSR II flow cytometer at the Albert Einstein College of Medicine (Bronx, NY).

### Quantitative PCR and RT-PCR

Wing discs were dissected from 15 WT (4 days AED) or 30 *hsRNZ*-rescued *RNZ^ΔMTS^* (7 or 14 days AED) third-instar larvae. For quantitative PCR, total DNA was purified using DNeasy Blood & Tissue Kit (Qiagen). For RT-PCR, total RNA was purified using NucleoSpin RNA II (Macherey–Nagel) and reverse transcribed with Transcriptor (Roche) using oligo(dT)_15_ or random hexamer primers. Quantitative PCR was performed with FastStart Universal SYBR Green Master (Roche) and Applied Biosystems 7300 Realtime PCR system as previously described (15). The efficiency of each primer set was determined using standard curves generated from 10-fold dilutions of control DNA/cDNA sample. The template levels of target DNA or cDNA were normalized to *Hsp27* or *rp49*, respectively. Primers are listed in the Supplementary Table S1. All qPCR assays were run in triplicate, and similar results were obtained from two independent experiments.

### Cellular ATP measurement

Imaginal discs from 15 WT (4 days AED) or hsRNZ rescued *RNZ^ΔMTS^* (14 days AED) larvae were dissected. Cellular ATP was extracted as described in (18), and quantified with ATP Measurement Kit (Invitrogen) using GloMax Luminometer (Promega). ATP concentrations were calculated from an ATP standard curve and normalized to total protein content.

### Cellular Lactate determination

Fifteen WT and 45 *RNZ^ΔMTS^* larvae were collected on fourth and seventh days AED, respectively, homogenized in cold PBS and centrifuged. 50-μl aliquots of supernatant were assayed using Lactate (Liquid) Reagent Set (Pointe Scientific). Absorbance was measured at 550 nm with Smartspec Plus Spectrophotometer (Bio-Rad). Lactate concentrations were calculated according to the standard curve and normalized to total protein content.

### Mitochondrial enzyme activity assays

Mitochondrial preparation, complex I activity assay and citrate synthase activity assay were performed as previously described (19). Mitochondria were prepared from 15 WT (4 days AED) and 60 *RNZ^ΔMTS^* (7 days AED) larvae.

### Mitochondrial ATP production

Mitochondrial ATP synthesis was measured in fresh mitochondrial preparations as described in (20). ATP production was normalized to total protein content.

Error bars from all biochemical assays indicate standard deviation of triplicate measurements from at least two independent experiments.

## RESULTS

### The amino terminus of Drosophila RNase Z^L^ contains MTS

**Figure 1.**
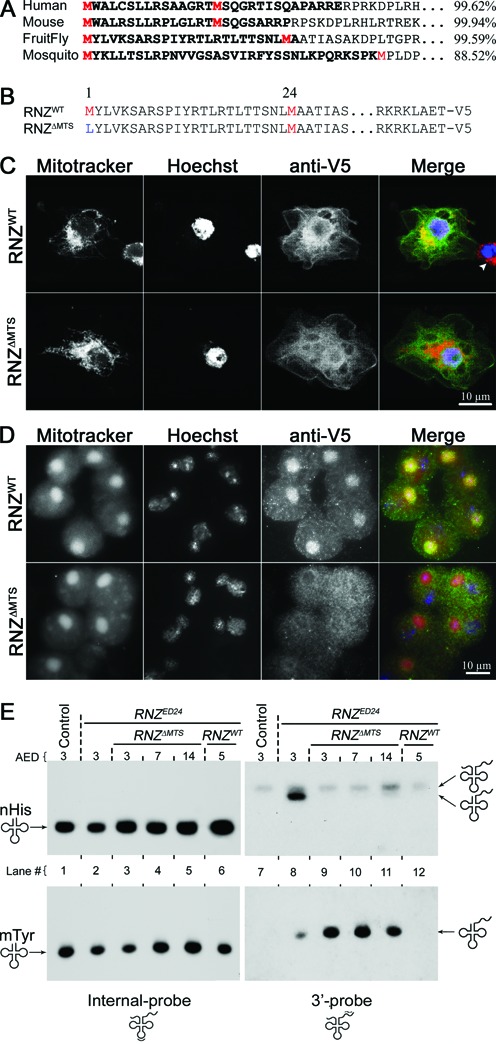
Mitochondria-specific knockout of *dRNaseZ*. (**A**) N-terminal sequences of RNase Z^L^ from different species. The mitochondria import probabilities (right) were calculated using MitoProt. Two initiating methionines are in red. The putative MTS is in bold. (**B**) The N-termini and C-termini of proteins encoded by *RNZ^WT^* and *RNZ^ΔMTS^* constructs. Methionines are in red and the mutated amino acid is in blue. V5-tags are attached to the C-termini of WT and mutant proteins. (**C**,**D**) Immunostaining of S2 cultured cells (C) and testes (D) expressing RNZ^WT^ and RNZ^ΔMTS^ proteins. In merge: mitochondria (Mitotracker) are red, DNA (Hoechst) is blue, and anti-V5 is green. (**C**) S2 cells transfected with plasmids encoding RNZ^WT^ or RNZ^ΔMTS^ proteins. Arrowhead indicates a non-transfected cell. (**D**) Onion stage spermatids from transgenic males carrying the *genRNZ^WT^* or *genRNZ^ΔMTS^* construct. Note that in (C,D) RNZ^WT^ co-localizes with mitochondria (yellow), while RNZ^ΔMTS^ does not. (**E**) Northern blot analysis of RNA samples from WT control (lane 1 and 7), *RNZ^ED24^* KO (lane 2 and 8) and *RNZ^ΔMTS^* (lane 3–5 and 9–11), or *RNZ^WT^* (lane 6 and 12) rescued KO larvae. The internal-probe detects mature nuclear Histidine (nHis) and mitochondrial Tyrosine (mtTyr) tRNAs; the 3′-probe detects primary transcripts and processing intermediates.

Our previous data on mitochondrial tRNA maturation imply presence of dRNaseZ in mitochondria. *In silico* analysis with MitoProt II detected a putative mitochondria-targeting signal (MTS) at the amino-terminus of dRNaseZ. Similar to homologs from other species (Figure 1A), the *Drosophila* MTS is flanked by two in-frame methionine codons, hinting that alternative initiation of translation may regulate the subcellular localization of dRNaseZ. To investigate the function of the putative MTS, we designed two constructs: *RNZ^WT^-V5* that encodes a wild-type (WT) protein tagged with the V5 epitope, and *RNZ^ΔMTS^-V5* that encodes the same protein except for the first methionine being changed to leucine, which forces the translation to start exclusively from the second initiation codon (Figure 1B). These constructs were transfected into S2 cells and the subcellular distribution of ectopic proteins was revealed with the anti-V5 immunofluorescent staining. Cells expressing RNZ^WT^ display its presence in all compartments – nuclei, cytosol and mitochondria. Cells expressing protein deprived of the MTS, RNZ^ΔMTS^, still have it in nuclei and cytosol, but lose its enrichment in mitochondria (Figure 1C).

To further confirm the mitochondrial localization of dRNaseZ *in vivo*, we created transgenic flies expressing RNZ^WT^ and RNZ^ΔMTS^ proteins under control of native dRNaseZ promoter (*genRNZ^WT^* and *genRNZ^ΔMTS^*). *Drosophila* spermatogenesis offers a convenient system to study the mitochondrial localization. At the end of meiosis II, mitochondria aggregate next to each haploid nucleus and then fuse into a spherical structure called Nebenkern. We found that the RNZ^WT^ protein is concentrated in the Nebenkern, while RNZ^ΔMTS^ is excluded from this mitochondrial formation (Figure 1D). These results demonstrate that the amino-terminal peptide sandwiched between two methionines is the functional MTS required for the mitochondrial targeting of dRNaseZ.

### A tool to dissect the mitochondrial function of dRNaseZ

Previous studies indicate that RNase Z^L^ has multiple duties in the nucleus, cytosol or mitochondria, some of which may not even require enzymatic activity (21–24). The *genRNZ^ΔMTS^* transgene encoding a protein devoid of MTS provides a genetic tool separating mitochondrial and other compartment functions of dRNaseZ. To study dRNaseZ activity specifically in mitochondria, we introduced *genRNZ^WT^* and *genRNZ^ΔMTS^* transgenes into the *dRNaseZ* null background *(RNZ^ED24^*). Thus, transgenes become the sole source of dRNaseZ proteins. We found that only *genRNZ^WT^* fully rescues the viability of the KO mutant (Supplementary Figure S1). The *genRNZ^ΔMTS^* transgene produces dRNaseZ without MTS. After hatching, *RNZ^ΔMTS^* larvae (*RNZ^ED24^;genRNZ^ΔMTS^*) grew and developed synchronously with heterozygous siblings (*RNZ^ED24^/+;genRNZ^ΔMTS^*), however, as they reached third instar, their growth was arrested. These larvae continued to crawl actively through food for another 3–4 weeks before they died without reaching full third instar size and possessing rudimentary imaginal discs.

To confirm that *RNZ^ΔMTS^* larvae specifically lack the mitochondrial activity of dRNaseZ, we examined the nuclear and mitochondrial tRNA processing in *RNZ^ED24^* in the absence and presence of either rescue constructs *genRNZ^ΔMTS^* and *genRNZ^WT^*. We performed a Northern blot analysis of nuclear tRNA^His^ and mitochondrial tRNA^Tyr^. To follow the tRNA processing, we designed two types of probes: the internal probes, complementary to the anticodon domain, reveal the steady state levels of tRNA; the 3′ probes, complementary to the 3′ trailer, reveal levels of tRNA gene expression. Internal probes for nucl-tRNA^His^ and mito-tRNA^Tyr^ detected single bands of comparable intensity indicating similar levels of mature tRNA in all samples (Figure 1E, lanes 1–6). In contrast, 3′ probes detected bands of processing intermediates, tRNA molecules with an extension at the 3′ end, in *RNZ^ED24^* (Figure 1E, lane 8). The *genRNZ^ΔMTS^* transgene rescued nuclear processing of nucl-tRNA^His^, but not mitochondrial of mito-tRNA^Tyr^ (Figure 1E, lanes 9–11). The *genRNZ^WT^* transgene rescued tRNA processing in both nuclei and mitochondria (Figure 1E, lane 12). These results validate the *RNZ^ΔMTS^* stock as a genetic tool to dissect the mitochondrial function of dRNaseZ.

### dRNaseZ is required for polycistronic transcript processing in mitochondria

**Figure 2.**
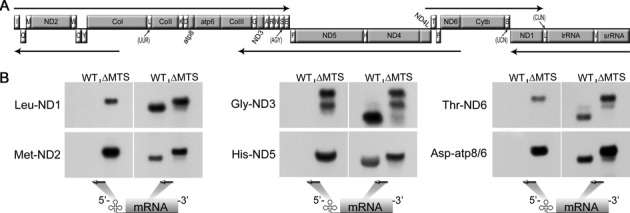
Mitochondrial transcript processing is affected in *RNZ^ΔMTS^* larvae. (**A**) Schematic representation of *Drosophila melanogaster* mitochondrial genome. Protein coding and ribosomal genes are designated by the gray boxes, and tRNAs (marked according to the single letter amino acid code) are designated by the white boxes. Arrows illustrate polycistronic transcripts (Torres *et al*., 2009). (**B**) Northern blot analysis of total RNA samples prepared from WT (5 days AED) and *RNZ^ΔMTS^* (7 days AED) larvae. Probes against tRNA and mRNA are shown as arrows. *RNZ^ΔMTS^* lanes display accumulation of processing intermediate (tRNA–mRNA) and absence of mature mRNA bands.

As mito-*tRNA* genes have a punctuation function in mtDNA (5), inability to process tRNA 3′ ends may compromise expression of other mitochondrial genes (Figure 2A). We used Northern blot hybridization to study mitochondrial primary transcript processing in *RNZ^ΔMTS^* larvae. With probes designed against tRNAs, we found significant accumulation of unprocessed transcripts identified as bicistronic intermediates – Leu-ND1, Met-ND2, Gly-ND3, His-ND5, Thr-ND6 and Asp-atp8/6 (Figure 2B). Bands of sizes bigger than bicistronic intermediates were never detected indicating that the damage is specific to the tRNA–mRNA junctions. With probes against mRNAs, we confirmed the identity of accumulated intermediates, and also found that levels of mature mRNAs drop precipitously in *RNZ^ΔMTS^* mutant. Thus, processing of mitochondrial mRNA transcripts that follow the neighboring tRNA depends on dRNaseZ activity.

### *RNZ^ΔMTS^* cells are defective in cell cycle progression

*RNZ^ΔMTS^* larvae possess barely identifiable rudimentary imaginal discs. However, one pulse of heat shock (HS) driven expression of the wild-type *dRNaseZ* transgene (*hsRNZ*) is sufficient to partially rescue growth of *RNZ^ΔMTS^* imaginal disc to a manageable size by day 7 AED. Those discs were dissected and tested for cell proliferation. Both markers of cell cycle—BrdU for S phase and phosphohistone H3 (PH3) for M phase—were severely reduced in wing and eye imaginal discs of *RNZ^ΔMTS^* larvae suggesting defects in cell cycle progression (Supplementary Figure S2).

**Figure 3.**
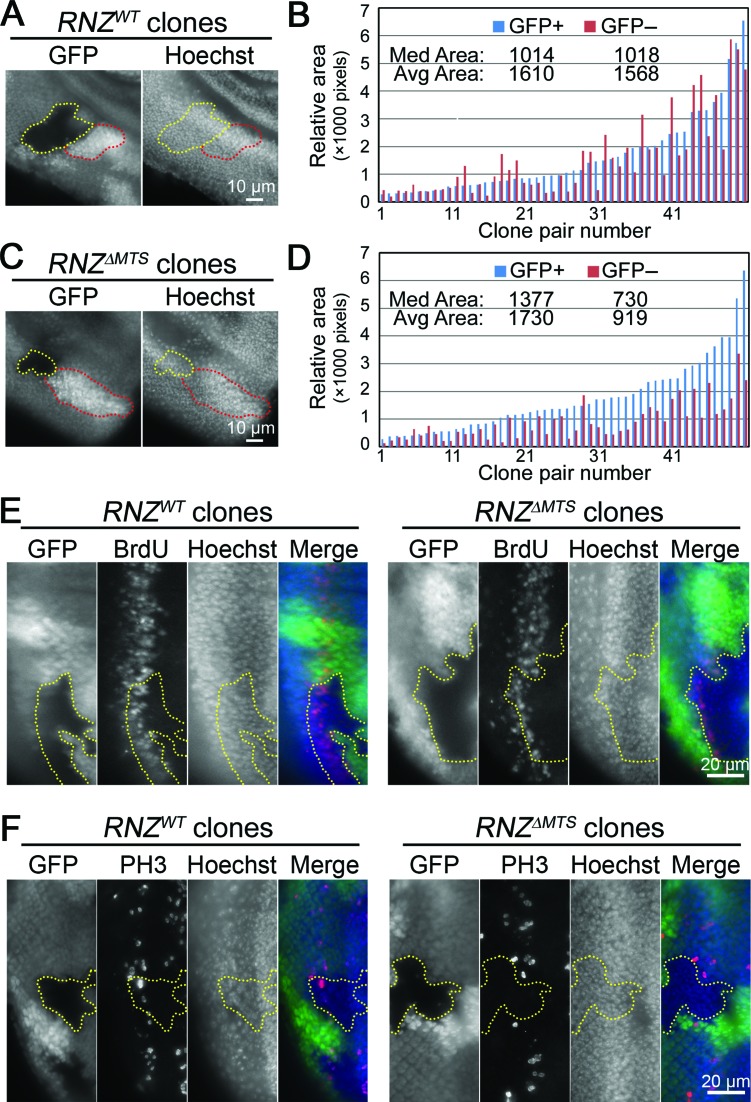
Mitochondrial dRNaseZ is autonomously required for imaginal disc cell proliferation. (**A**,**C**) Fluorescent images of third instar eye discs containing *RNZ^WT^* and *RNZ^ΔMTS^* clones in a wild-type background. Yellow dots outline *RNZ^WT^* and *RNZ^ΔMTS^* clones (GFP negative); red dotes outline the wild-type twin-spots (double GFP). Nuclei are stained with Hoechst. (**B**,**D**) Areas of clones and corresponding twin-spots measured in pixels with histogram function of Adobe Photoshop. Bars are ordered on the X-axis according to the size of the twin-spot (blue, GFP+). Values for median and average clone areas are indicated. *RNZ^WT^* clones (B) have similar sizes with wild-type twin-spots (*P* = 0.67), while *RNZ^ΔMTS^* clones (D) are smaller (*P* < 0.0001, *t*-test). (**E**,**F**) BrdU incorporation (E) and PH3 staining (F) posterior of the morphogenetic furrow in third instar eye discs. In merge: GFP, green; BrdU or PH3, red; Hoechst, blue. GFP negative *RNZ^WT^* and *RNZ^ΔMTS^* clones are outlined with yellow dots, and discs are oriented with anterior to the right. Note that *RNZ^ΔMTS^* clones have thinner BrdU band and no PH3 staining.

To test whether imaginal disc growth deficiency is cell-autonomous, we used the FLP/FRT system. Mitotic clones, homozygous for the *RNZ^ED24^* null allele, were generated either in the *genRNZ^WT^* or *genRNZ^ΔMTS^* genetic background. By comparing sizes of clones and corresponding twin spots, we found that unlike wild-type cells, *RNZ^ΔMTS^* produced smaller clones suggesting a cell-autonomous damage in proliferation (Figure 3A–D). To confirm this conclusion, we studied BrdU incorporation and anti-PH3 staining in mosaic eye imaginal discs. We generated *RNZ^ΔMTS^* and wild-type clones posterior to the morphogenetic furrow, where a band of cells synchronously enter the final round of mitosis. While all cells within this band, including cells of *RNZ^WT^* clones, are BrdU-positive, *RNZ^ΔMTS^* clones have a thinner BrdU band, indicating fewer cells in S phase (Figure 3E). With the anti-PH3 antibody we did not find any *RNZ^ΔMTS^* cells in M phase, while *RNZ^WT^* clones revealed a normal pattern of PH3 staining (Figure 3F). These data confirm that the KO of mitochondrial dRNaseZ breaks cell cycle progression in a cell-autonomous fashion.

**Figure 4.**
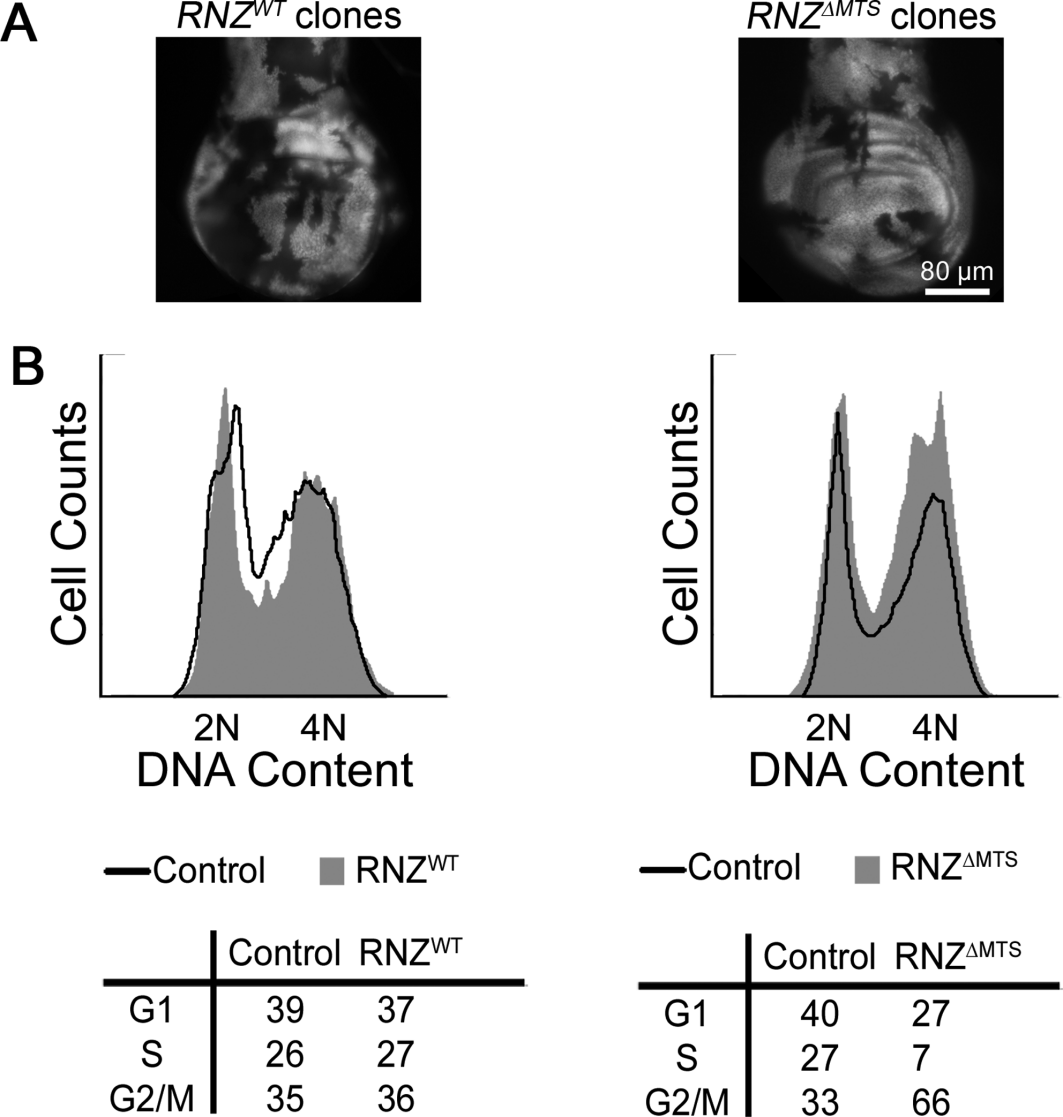
*RNZ^ΔMTS^* cells are delayed at the G_2_/M transition. (**A**) Representative third instar wing discs containing *RNZ^WT^* and *RNZ^ΔMTS^* clones (GFP negative) in a *Minute* background. (**B**) Flow cytometry analysis of wing discs containing *RNZ^WT^* (left) and *RNZ^ΔMTS^* (right) clones. In the histogram, the X-axis represents relative DNA content and the Y-axis represents cell count. Black traces correspond to the GFP-positive control and gray filled traces correspond to the GFP-negative experimental groups. Tables display the percentage of cells in the G1, S and G2/M phases in each group tested. *RNZ^ΔMTS^* has lower proportion of cells in G1 and S phases, and higher proportion of cells in G2/M.

To improve the yield of *RNZ^ΔMTS^* cells for flow cytometry analysis, we used the *Minute* genetic background. Heterozygous *M*/*+* cells are delayed in their development and thus create less competitive environment to facilitate growth of homozygous mutant cells. Indeed, induction of mitotic recombination in the *M*/*+* background generated larger *RNZ^ΔMTS^* clones, although not as large as wild-type clones (Figure 4A). Wing discs containing mitotic clones were dissociated and analyzed by flow cytometry for cell cycle phasing. We found that in control discs with *RNZ^WT^* clones, GFP-negative and GFP-positive cells have similar cell cycle profiles. In discs with *RNZ^ΔMTS^* clones, mutant cells are less abundant in G1 and S, but accumulate in G_2_/M (Figure 4B). The average size of *RNZ^ΔMTS^* cells is similar to *M*/*+* neighbors suggesting that their growth is not affected (Supplementary Figure S3). We also confirmed the G_2_/M accumulation of *RNZ^ΔMTS^* cells using mutant discs dissected from *hsRNZ*-rescued larvae (Supplementary Figure S4). Together, these data indicate that mitochondrial dRNaseZ activity is required for the G_2_/M transition.

### *RNZ^ΔMTS^* cells show a decrease in mitochondrial activity and increase in ROS levels

**Figure 5.**
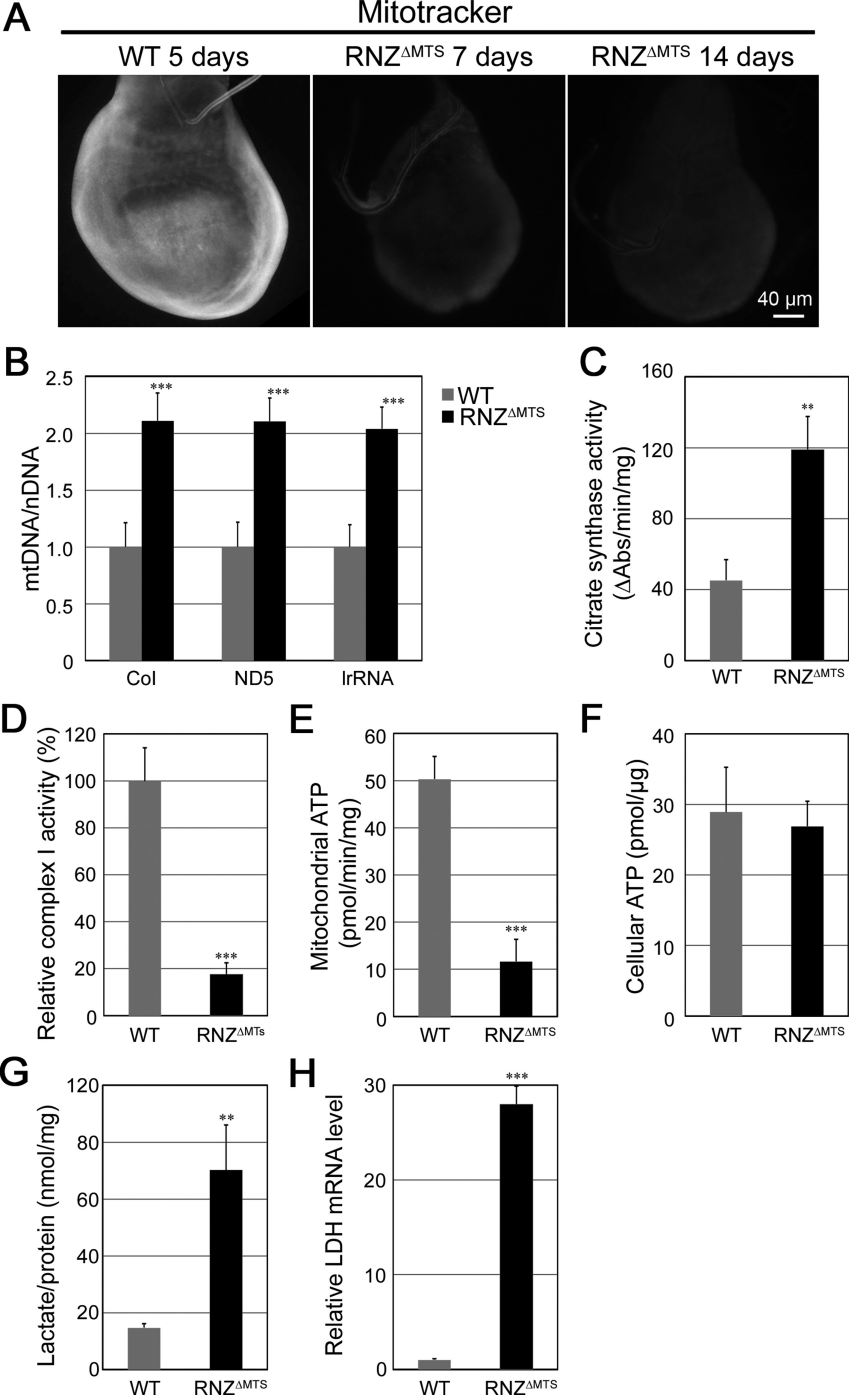
*RNZ^ΔMTS^* larvae have decreased mitochondrial activity. (**A**) *In vivo* Mitotracker staining of third instar wing discs dissected from WT and *hsRNZ*-rescued *RNZ^ΔMTS^* larvae. (**B**) qPCR analysis of mtDNA in WT and *hsRNZ*-rescued *RNZ^ΔMTS^* wing discs. mtDNA values (*CoI, ND5, lrRNA*) are normalized to nuclear DNA (*Hsp27*). The value of WT mtDNA is set as 1. Citrate synthase activity (**C**), complex I activity (**D**), and ATP synthesis capacity (**E**) are measured in mitochondrial preparations obtained from WT and *RNZ^ΔMTS^* larvae. Complex I activity is normalized to citrate synthase activity. (**F**) Cellular ATP content from WT and *hsRNZ*-rescued *RNZ^ΔMTS^* wing discs. (**G**) Lactate levels in extracts from WT and *RNZ^ΔMTS^* larvae. (H) qRT-PCR analysis of *Ldh* expression in WT and *RNZ^ΔMTS^* wing discs. Transcript levels are normalized to rp49. Error bars indicate standard deviations. **P < 0.01, ***P < 0.001, two-tailed *t*-test.

Consistent with the observation that mitochondrial *dRNaseZ* KO compromises expression of genes encoding subunits of the respiratory chain, we also found that it disrupts the mitochondrial membrane potential as evidenced by a strong reduction of MTRed staining in *RNZ^ΔMTS^* imaginal discs (Figure 5A). Next, we tested whether mitochondrial biogenesis and content were affected by the loss of dRNaseZ. Real-time qPCR analysis of mtDNA per cell showed at least a 2-fold increase in *RNZ^ΔMTS^* discs (Figure 5B). Activity of citrate synthase, an enzyme marker of mitochondrial mass, was twice as high in mutant larvae as in control (Figure 5C). These data suggest that *dRNaseZ* KO impairs the OXPHOS system and initiates compensatory mitochondrial biogenesis.

To get a quantitative measure of OXPHOS performance, we conducted a biochemical test of ETC complex I and found an 80% drop of its activity in *RNZ^ΔMTS^* larvae (Figure 5D). The ATP synthesis capacity of *RNZ^ΔMTS^* mitochondria was also down 5-fold (Figure 5E). To our surprise, when we measured the steady-state cellular ATP levels in *RNZ^ΔMTS^* and control wing discs we did not find a statistically significant difference (Figure 5F). We hypothesized that in order to maintaining normal ATP levels mutant cells could switch from OXPHOS to glycolysis. When we measured concentration of lactate, as an indicator of glycolytic pathway, we found that it is about 5-fold higher in *RNZ^ΔMTS^* than in control larvae (Figure 5G). Expression of the gene encoding lactate dehydrogenase (LDH), the enzyme that converts pyruvate into lactate and maintains glycolytic flux, is also up about 30-fold (Figure 5H). These data clearly indicate a switch toward glycolysis as a compensatory pathway of ATP production in cells of *RNZ^ΔMTS^* larvae.

**Figure 6.**
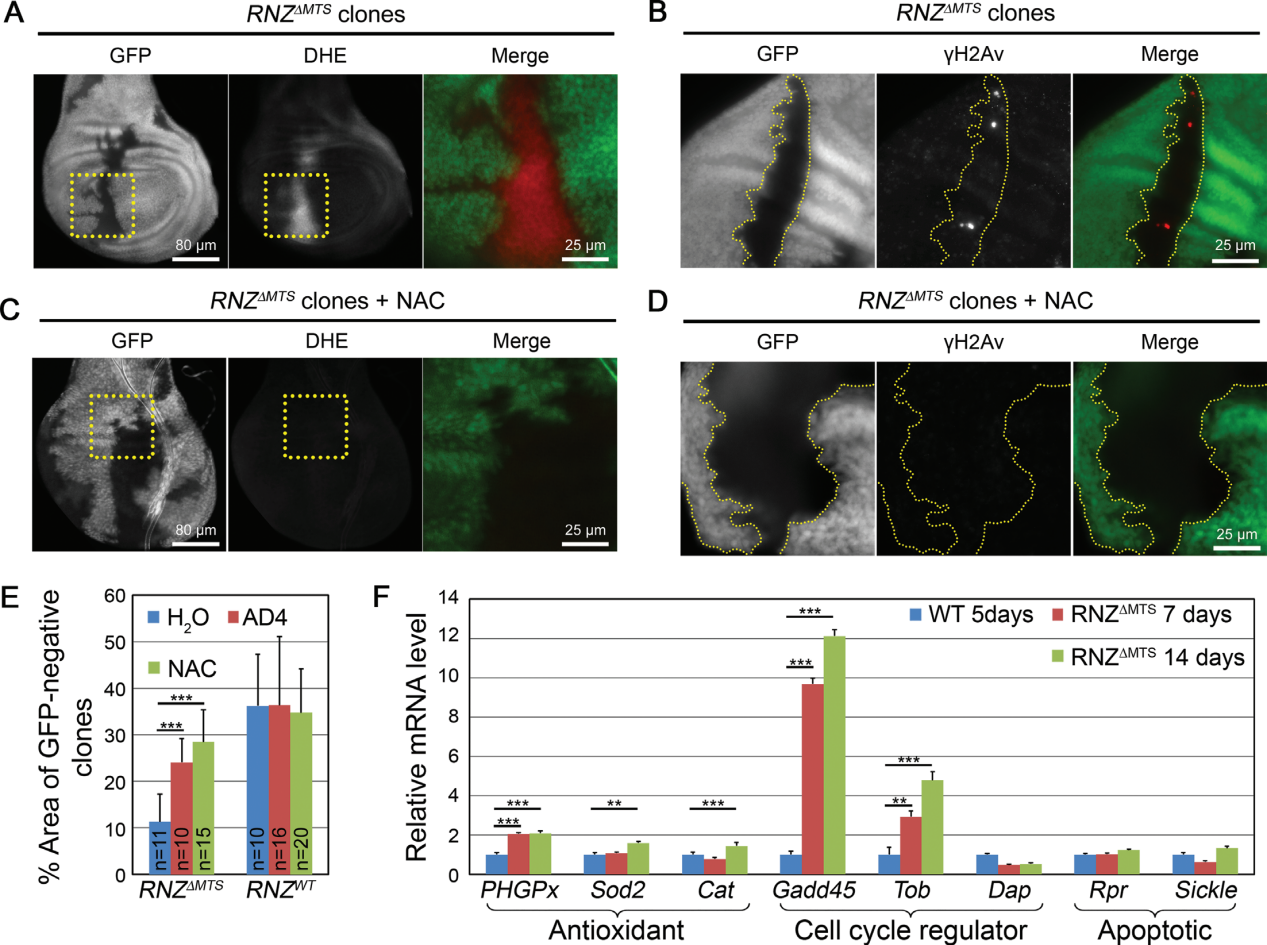
*RNZ^ΔMTS^* cells accumulate ROS leading to the genotoxic stress and cell cycle delay. (**A**–**D**) *RNZ^ΔMTS^* clones generated on *Minute* background are marked by the absence of GFP. (**A**,**C**) Dihydroethidium (DHE) staining (red in merge) of untreated and NAC treated wing discs with *RNZ^ΔMTS^* clones. Regions bound by dashed yellow boxes are shown in higher magnification on the *right* as merged color image. (**B**,**D**) γH2Av labeling (red in merge) of untreated and NAC treated wing disc with *RNZ^ΔMTS^* clones. GFP negative *RNZ^ΔMTS^* clones are outlined by yellow dots. NAC reduces ROS and γH2Av staining in mutant clones. (**E**) Quantification of the area occupied by *RNZ^ΔMTS^* or *RNZ^WT^* clones (GFP negative) relative to total wing disc area. NAC and AD4 increase the percentage area occupied by *RNZ^ΔMTS^* clones, while have no effect on discs containing *RNZ^WT^* clones. (**F**) qRT-PCR analysis of p53 target genes expressed in WT and *hsRNZ*-rescued *RNZ^ΔMTS^* wing discs. Transcript levels are normalized to rp49. Error bars indicate standard deviations. **P < 0.01; ***P < 0.001, two-tailed *t*-test.

As impairments of ETC activity often lead to increased production of ROS, we studied their intracellular levels using dihydroethidium (DHE) dye in *RNZ^ΔMTS^* mosaic imaginal discs. Cells without mitochondrial dRNaseZ displayed a sharp increase in ROS content compared to wild-type neighboring cells (Figure 6A). We observed the same strong accumulation of ROS in *RNZ^ΔMTS^* conditionally rescued larvae (Supplementary Figure S5). Thus, the KO of mitochondrial dRNaseZ leads to respiratory chain dysfunction and increased ROS levels that may affect cell proliferation.

### ROS and genotoxic stress

Cells accumulating ROS may suffer from a genotoxic stress associated with a range of nuclear DNA lesions such as base modifications and single- or double-strand breaks (DSB). Phosphorylation of the histone variant H2Av is the earliest event after DSB formation (25), and thus could serve as a marker for DNA damage. To see if *RNZ^ΔMTS^* cells experience genotoxic stress, we examined mosaic imaginal discs with the antibody specific to a phosphorylated form of histone H2Av (γH2Av). We found a number of γH2Av foci of different intensity within *RNZ^ΔMTS^* clones, but not in the neighboring control cells (Figure 6B and Supplementary Figure S6A). Western blot assay confirmed accumulation of phosphorylated H2Av in *RNZ^ΔMTS^* wing discs (Supplementary Figure S6B). These data indicate that the KO of mitochondrial *dRNaseZ* results in a genotoxic stress.

As excessive DNA damage could induce cell death, we stained mosaic imaginal discs with antibody specific to activated caspase-3 (Casp3), an apoptotic marker. We did not find caspase-3 activation in mutant clones, indicating that loss of mitochondrial *dRNaseZ* does not lead to apoptosis (Supplementary Figure S7).

Based on these data, we proposed that in *RNZ^ΔMTS^* cells ROS induce a cell survival genotoxic response with a cell cycle delay allowing time for DNA repair. To test this interpretation, we attempted a rescue of impaired cell proliferation by decreasing ROS levels with widely used anti-oxidant reagents *N*-acetylcysteine (NAC) and *N*-acetylcysteine amide (AD4). First, we confirmed that NAC treatment efficiently reduced intracellular ROS levels (Figure 6C) and rescued DNA damage in *RNZ^ΔMTS^* clones (Figure 6D and Supplementary Figure S6A). Next, we examined the size of *RNZ^ΔMTS^* clones in mosaic imaginal discs dissected from larvae grown on regular food or the one supplied with the antioxidant. Both AD4 and NAC had a strong positive effect on cell proliferation (Figure 6E), as the overall area occupied by GFP-negative *RNZ^ΔMTS^* cells increased from 11% ± 6% (before antioxidant treatment) to 24% ± 5% and 28% ± 7% (after AD4 and NAC treatments, respectively). We note that antioxidants did not produce a complete rescue, as WT clones generated in parallel were consistently larger and occupied 36% ± 10% of disc area (Figure 6E). Overall, our results indicate that cells deficient for dRNaseZ mitochondrial activity accumulate ROS causing genotoxic stress that does not affect cell viability, but produce a cell cycle arrest.

### Coordinated oxidative stress response

Given that tumor suppressor p53 is a known sensor of oxidative stress, we hypothesized that in *RNZ^ΔMTS^* cells ROS activate cell cycle delay through the p53 pathway. The p53 protein is a transcription factor, so its activation could be defined by the transcriptional profiling of p53 target genes. In general, p53 can induce diverse responses ranging from survival to apoptosis. Genome-wide analyses with ChIP-exo and GRO-sec identified over 100 genes comprising the direct p53 transcriptome (26,27). From this complex network, we selected eight target genes that are known to be activated by endogenous ROS and represent distinct stress responses. First group includes the antioxidant defense enzymes: cytosolic catalase (Cat), mitochondrial superoxide dismutase (Sod2), and mitochondrial phospholipid hydroperoxide glutathione peroxidase (PHGPx). Second group is cell cycle regulators: Gadd45 and Tob are activators of the G_2_/M checkpoint, they are cycB-Cdk1 inhibitors; and p21/Dap is a cyclin-dependent kinase inhibitor necessary for the G_1_/S checkpoint. And third group is two activators of apoptosis: Reaper (Rpr) and Sickle (Skl). By choosing this subset of p53 direct targets, we expected to develop an understanding how cell fate choice in proliferating imaginal discs is defined upon mitochondrial dRNaseZ KO.

The expression of eight genes was examined by qRT–PCR with total RNA isolated from control and *RNZ^ΔMTS^* wing discs (Figure 6F). Upon mitochondrial *dRNaseZ* knockout, *Gadd45* and *Tob* genes respond with strong upregulation of 12- and 5-fold, respectively, while expression of the G_1_/S inhibitor *p21*/*Dap* is not activated. Transcripts of the antioxidant genes, *PHGPx*, *Sod2* and *Cat*, show modest accumulation of 1.5- to 2-fold. Expression of the apoptotic genes, *Rpr* and *Skl*, does not change. Thus, the knockout of mitochondrial *dRNaseZ* activity generates a condition of modest stress that does not affect cell viability, but bolsters antioxidant defense and brings about a cell cycle delay by activating the G_2_/M checkpoint.

## DISCUSSION

Here, we describe a mitotic checkpoint that is activated *in vivo* as a result of mitochondrial distress caused by an organelle-specific knockout of dRNaseZ, a nuclear-encoded enzyme required for mitochondrial transcript processing.

### A vital role of dRNaseZ in mitochondria

*Drosophila* RNase Z^L^ is a highly conserved protein with orthologs in all eukaryotes. Mutations in *ELAC2*, a human gene encoding *RNase Z^L^*, have been associated with the occurrence of prostate cancer (PCA) and infantile hypertrophic cardiomyopathy (HCM) (28,29). However, it is not clear what role sequence variants of ELAC2/RNase Z^L^ may play in the genesis of human pathologies. Analysis of RNase Z^L^ is challenged by the multifuctionality of the protein, as besides processing pre-tRNA, it has been proposed to regulate cell cycle and gene expression (21,23). In terms of the subcellular localization, RNase Z^L^ endonucleolytic activity has been detected in nucleus, mitochondrion and cytosol, generating a spectrum of products including tRNA, rRNA, miRNA and tRF (22,24,30). The amino termini of RNase Z^L^ contain putative MTS; human ELAC2/RNase Z^L^ with this targeting signal can localize to the mitochondria in cultured cells (31). Our study shows, for the first time *in vivo*, that the endogenous RNase Z^L^ without MTS is excluded from the mitochondria (Figure 1C and D). Similar to its mammalian counterpart, dRNaseZ has its MTS sandwiched between two methionines implying that translation from alternative AUG codons yields two protein forms – the long one is mitochondrial, and the short one may localize in the nucleus or cytosol. Taking advantage of these naturally occurring alternative initiation codons, we developed a genetic tool, *RNZ^ΔMTS^*, to study the mitochondrial function of *Drosophila* RNase Z^L^. Using tRNA processing as a test we confirmed that *RNZ^ΔMTS^* larvae possess a fully functional short form of dRNaseZ, but not the long form, as they accumulate only mitochondrial unprocessed tRNAs with 3′ extensions (Figure 1E). The KO of mitochondrial dRNaseZ is lethal; *RNZ^ΔMTS^* larvae develop into third instar, but they never grow to the full size. Growth arrest due to dRNaseZ deprivation in mitochondria was expected, and reflects a general requirement of functional mitochondria for growth control.

### Mitochondrial dRNaseZ KO results in a metabolic shift and oxidative stress

Similar to *RNZ^ΔMTS^*, knockout of many mitochondrial proteins impairs animal growth (32,33). It is not surprising that mutant growth arrest is often caused by cell cycle defects (34), as bioenergetics and proliferation are cellular functions that are intricately intertwined. It has been found that energy management changes with the cell cycle progression. In G_1_ phase, proliferating cells primarily rely on accelerated glycolysis, and in G_2_/M – on mitochondrial respiration (35). Transition to OXPHOS depends on kinase activity of CycB1/Cdk1, a fraction of which relocates to mitochondrial matrix and elevates ATP production at G_2_ (36). We found that dRNaseZ is required to support aerobic respiration and G_2_/M transition. It processes mitochondrial polycistronic transcripts yielding mRNAs for OXPHOS subunits. In *RNZ^ΔMTS^* cells, transcripts for complexes I and V are strongly affected (Figure 2B). Consistently, one of the main electron entry points to the ETC, complex I, and the ATP synthase, complex V, are disabled (Figure 5D and E). Remarkably, *RNZ^ΔMTS^* mutant reprograms cells to the glycolytic pathway to supply all their ATP demands (Figure 5F). The metabolic switch is evidenced by lactate accumulation and increased expression of the *Ldh* gene (Figure 5G and H). Curiously, a shift to aerobic glycolysis, known as the Warburg effect, is among main characteristics of cancer including PCA, and elevated serum lactate levels, or lactic acidosis, is one of the clinical features in individuals with severe HCM. Our data suggest that these pathophysiological conditions found in patients with *ELAC2* mutations might be due to reduction in mitochondrial RNase Z^L^ activity.

Mitochondrion is the major source of ROS, as even under normal electron flow, ETC leaks a small percentage of electrons to oxygen converting it into superoxide. KO of mitochondrial dRNaseZ disables ETC and increases ROS generation. Once become excessively high, ROS could be toxic, damaging cellular biopolymers and causing cell death. However, it is well documented that at sublethal levels, ROS are signaling molecules modulating cell cycle, cell differentiation, stem cell self-renewal and cell signaling (37–39). In *RNZ^ΔMTS^* cells, excess ROS produce genotoxic stress and force the G_2_/M cell cycle delay. However, they do not affect cell viability. We suggest that in cells lacking mitochondrial dRNaseZ, ROS trigger a pro-survival program. Instead of permanent cell cycle arrest or apoptosis, stressed cells are engaged in repair pathways. *RNZ^ΔMTS^* cells display an increased phosphorylation of H2Av indicating activation of the ATM kinase-dependent DNA repair pathway (Figure 6B). Cell cycle delay allows time for the DNA repair machinery to fix damage and maintain cell viability. Metabolic reprogramming of *RNZ^ΔMTS^* cells could also be part of the anti-stress program that maintains ROS at sublethal level. Transition to aerobic glycolysis down-regulates tricarboxylic acid cycle and restricts the electron flux through ETC, thereby decreasing ROS production (40). Moreover, glycolysis enhances antioxidant defense by supplying pyruvate and lactate scavenging free radicals (41,42). Our finding that loss of mitochondrial dRNaseZ leads to ROS accumulation together with increased glycolysis and cell cycle delay suggests that powerless mitochondria initiate retrograde signaling stimulating cellular responses to overcome their flawed state.

### Retrograde signaling in cells void of mitochondrial dRNaseZ

**Figure 7.**
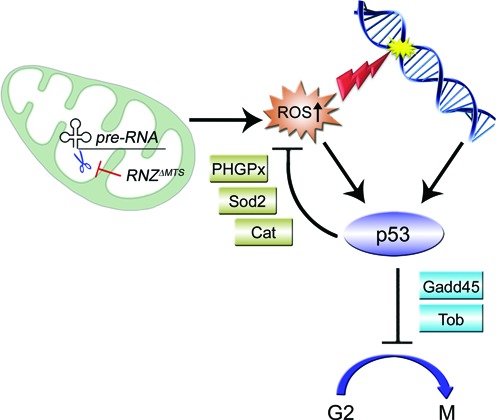
Proposed mechanism for retrograde signaling that links mitochondrial transcript processing to cell proliferation. The impairment of primary transcript processing in mitochondria decreases abundance of mRNAs, encoding protein subunits of the respiratory chain. Inactivation of complexes I and V increases mitochondrial ROS formation. Accumulating ROS lead to oxidative stress with cellular and DNA damage. To withstand low-level oxidative stress, cells initiate a specific transcriptional response, which enforces the G2 checkpoint via the cycB-Cdk1 inhibitors Gadd45 and Tob, and promotes cell survival by lowering intracellular ROS levels and allowing DNA repair before mitosis.

Although the impact of dysfunctional mitochondria on cell proliferation has been documented, this is the first demonstration of a retrograde signaling pathway targeting the G_2_/M checkpoint. Figure 7 offers a model linking initial damage of polycistronic transcript processing to cell cycle arrest. As a supply of the dRNaseZ protein declines in *RNZ^ΔMTS^* cells, mitochondria become defective resulting in a loss of membrane potential and generation of ROS, such as superoxide anions from the ETC. Superoxide anions may directly diffuse from the intermembrane space into cytosol or first be converted into H_2_O_2_ and then get released from mitochondria. ROS are highly reactive molecules that can cause stress via oxidative damage to cell structures. In case the antioxidant defense enzymes, such as Cat, Sod2 or PHGPx, fail to control ROS levels, cells undergo ROS-induced apoptosis. On the other hand, when present at moderate or physiologically relevant levels, intracellular ROS participate in the retrograde signaling, as they activate downstream proteins, e.g. transcription factors, via oxidation of redox sensitive cysteine residues (43). We suggest that in *RNZ^ΔMTS^* cells, p53 is the downstream protein that upon activation enacts the antiproliferative program. As *RNZ^ΔMTS^* clones are smaller but readily visible in the twin spot analysis (Figure 3), and *RNZ^ΔMTS^* cells do not show signs of apoptosis (Supplementary Figure S7), we conclude that the KO of mitochondrial dRNaseZ generates a low-level stress. Cells respond with a cell cycle delay allowing time for the damage to be fixed before resuming proliferation. Many studies have shown that p53 functions as a stress sensor that can be activated either directly by ROS in a dose-dependent manner or indirectly by ATM and Chk2 kinases as a part of the DNA damage response (44). As a transcription factor, p53 binds and selectively modulates expression of a variety of genes, which may lead to diverse biological responses ranging from adaptive to cell death programs (45,46). Correspondingly, the p53-regulated genes can be divided into several functional sets, such as DNA repair, antioxidant, cell cycle regulating or pro-apoptotic genes. Analysis of a subset of p53 target genes (Figure 6F) activated in *RNZ^ΔMTS^* cells shows that oxidative stress initiates an adaptive response that enhances antioxidant defense with PHGPx, Sod2 and Cat enzymes, delays cell cycle via Gadd45 and Tob inhibitors of cycB-Cdk1, and promotes cell survival (Figure 7).

There are some other homeostatic or non-apoptotic outcomes after dRNaseZ KO where p53 might have a role complementary to its inhibitory effect on cell cycle progression. One of p53 potential functions is maintaining the integrity of mitochondrial genome. It has been shown that under conditions of a mild physiological stress a fraction of activated p53 relocates to mitochondria, where it physically interacts with the DNA polymerase γ enhancing mtDNA replication and repair (47). In *RNZ^ΔMTS^* cells, we indeed found a 2-fold increase of mtDNA content (Figure 5B), which suggests an improved Pol γ processivity and mitochondrial genome stability. Further experiments are required to confirm the direct role of p53 in mtDNA replication in *RNZ^ΔMTS^* cells.

Another pro-survival activity of p53 is metabolic regulation. Previous studies have suggested that p53 functions control aerobic respiration and glycolysis (48). In *RNZ^ΔMTS^* cells, we observed a sharp increase in lactate levels and *Ldh* gene expression (Figure 5G and H), which illustrate the metabolic switch from OXPHOS to aerobic glycolysis. Without additional experimentation, however, the role of p53 in this switch is hypothetical.

One observation in this study that stands out is a delay of cell cycle at the G_2_/M boundary caused by the KO of mitochondrial dRNaseZ. Cell cycle has two checkpoints G_1_/S and G_2_/M that are energy and DNA damage sensitive. Still, previous studies of mutations in nuclear genes that encode mitochondrial proteins—CoVa, PdsW, mRpL17, mRpL4—identified alterations only in the transition from G_1_ to S phase (34). The KO of dRNaseZ is the first perturbation of mitochondria that affects mitotic cell cycle progression at the G_2_ to M transition. We do not yet understand all the details of how mitochondria impact the G_2_ checkpoint, though we believe that ATP is not a factor, as cellular ATP levels are not decreased in *RNZ^ΔMTS^* larvae. The delay in cell cycle progression is mainly due to ROS overproduction and subsequent oxidative stress, as evidenced by its reversal following the antioxidant treatment (Figure 6C–E). Interestingly, we found that antioxidants did not completely restore the proliferative efficiency, as rescued *RNZ^ΔMTS^* clones were about 80% in size of WT clones. We propose that unlike previously described mutations, dRNaseZ KO produces a mitochondrial damage that is not necessarily more profound, but rather more complex. Besides ROS, *RNZ^ΔMTS^* cells accumulate other molecules with potential retrograde signaling activity, e.g. lactate and RNA processing intermediates. Also, because of defective mitochondrial transcript processing, *RNZ^ΔMTS^* cells may miss particular molecules such as noncoding RNAs that appear to play a role in cell cycle regulation (49). While each of these cases warrants a special study, we suggest that metabolites missing or accumulating in the *RNZ^ΔMTS^* cells could activate parallel signaling cascades or modulate ROS initiated pathway to produce the anti-proliferative stress response with the G_2_/M delay.

## Supplementary Material

SUPPLEMENTARY DATA
