# Supplementary material for: Knockout of Drosophila RNase ZL impairs mitochondrial transcript processing, respiration and cell cycle progression
Source: Nucleic Acids Res. 2015 Nov 8;43(21):10364–75. doi: 10.1093/nar/gkv1149 (PMC4666369; doi:10.1093/nar/gkv1149)
Supplement: SUPPLEMENTARY DATA [file supp_43_21_10364__index.html]

Knockout of Drosophila RNase ZL impairs mitochondrial transcript processing, respiration and cell cycle progression — SUPPLEMENTARY DATA 

# Knockout of *Drosophila* RNase ZL impairs mitochondrial transcript processing, respiration and cell cycle progression

## SUPPLEMENTARY DATA

- SUPPLEMENTARY DATA
