# Supplementary material for: Knockout of Drosophila RNase ZL impairs mitochondrial transcript processing, respiration and cell cycle progression
Source: Nucleic Acids Res. 2015 Nov 8;43(21):10364–75. doi: 10.1093/nar/gkv1149 (PMC4666369; doi:10.1093/nar/gkv1149)
Supplement: SUPPLEMENTARY DATA [file supp_gkv1149_nar-02479-v-2015-File009.pdf]

Supplemental tables

Table S1 Primers used in this study.

Primers for mutagenesis and plasmids:	
Name	Sequence (5'-3')
genRNZM1LF	CTCTGACGAAATACGA**T**TGTATTTAGTAAAATCAGCC
genRNZM1LR	GGCTGATTTTACTAAATACA**A**TCGTATTTCGTCAGAG
KpnI-RNZ	CGG**GGTACC**ATGTATTTAATAAAATCAGCC
XhoI-RNZ	**CTCGAG**TTTCTGCCAGTTTTCGC
pMTRNZM1LF	CGATTAGGGAATTGGGAATTC**T**TGTATTTAATAAAATCAGCCG
pMTRNZM1LR	CGGCTGATTTTATTAAATACA**A**GAATTCCCAATTCCCTAATCG

Primers for quantitative PCR:			
Target	FlyBase ID	Forward primer (5'-3')	Reverse primer (5'-3')
Hsp27	FBgn0001226	TTGCACTCTGAAAAGACAGCTTT	AAGCAAAACGAACAGTTAAAATGC
CoI	FBgn0000343	GGTGCTCCTGATATAGCATTCCCAC	ACAGTTCATCCTGTTCCAGCTCC
ND5	FBgn0013684	AAGAAGTAAAGCTACATCCCCAATTCG	GGGTGAGATGGTTTAGGACTTGTTTC
lrRNA	FBgn0013686	GAAACCAACCTGGCTTACACCG	GATTGCGACCTCGATGTTGGATTAAG
RP49	FBgn0002626	AAGAAGCGCACCAAGCAC	TTGGGCATCAGATACTGTCC
LDH	FBgn0001258	GCAACACCGACATCCTCAAG	CGGGATTGGACACCATAAGC
PHGPx	FBgn0035438	GTGTTCGCCAAGGTCGATG	AACTTGGTGAAGTTCCACTTG
Sod2	FBgn0010213	TTCGCAAACTGCAAGCCTGG	TGTTGACGTAGGTCTGGTGG
Cat	FBgn0000261	ACTACTTTGCTGAGGTGGAG	CGATGGGTGTCCGAGTAGG
Gadd45	FBgn0033153	GGGAGCAGAGATGGATAAGC	AGTATCTGTGCGCGACTTCC
Tob	FBgn0028397	TCCAAGCGCACAAACAGCAG	TGCCGATACCGATGATCCTG
Dap	FBgn0010316	GTCCTGCGCAAGAGACAGC	CGGACGAACTGGGAGAAC
Rpr	FBgn0011706	GGAAAACCAATAGTCCAGTCC	GGTGTTGTGGCTCTGTGTC
Grim	FBgn0015946	GGTGTTGTGGCTCTGTGTC	ATTCTTGTTGCTGCGGTTGC

Table S2 Probes used in this study.

tRNA	FlyBase Gene	Internal probe (5'-3')	3'-end probe sequence (5'-3')
nHis	FBgn0050251	GAACCTGGGTTACCACGGCCACAA	ACCCAACTCCGTGACAATGTTTGT
mtTyr	FBgn0013710	GCCTAAACTTCAGCCACTTA	AATTAATTTGTCCTTATTTG
mtLeu^{CUN}	FBgn0013698	AACAAGTACTATTTGTAATAAAAATCACA	
mtMet	FBgn0013700	GGGGTATGAACCCAGTAGCTTA	
mtGly	FBgn0013694	GACCTTATGATTGGAAGTCAAATATAC	
mtHis	FBgn0013695	GAATATTTTCATATCACTAACACCACAA	
mtAsp	FBgn0013691	GTTTGACATACTAATGTTATGATTTTAAC	
mtThr	FBgn0013707	GATTTACAAGACCAATGTTTTTATTAAAC	
mtND1	FBgn0013679	GAAATAGTCTGAGCCACAGCTCGCA	
mtND2	FBgn0013680	GCTCCTAACCAAGAATTAGATGTAAC	
mtND3	FBgn0013681	GGATCAAATCCACATTCAAATGGGG	
mtND5	FBgn0013684	GAAACAAGTCCTAAACCATCTCACC	
mtND6	FBgn0013685	TTCATTAGAGGCTAAAGATGTTACGTA	
mtATP6	FBgn0013672	TCGAACAGCTAATGTTCCAGGTCG	

Supplemental figures

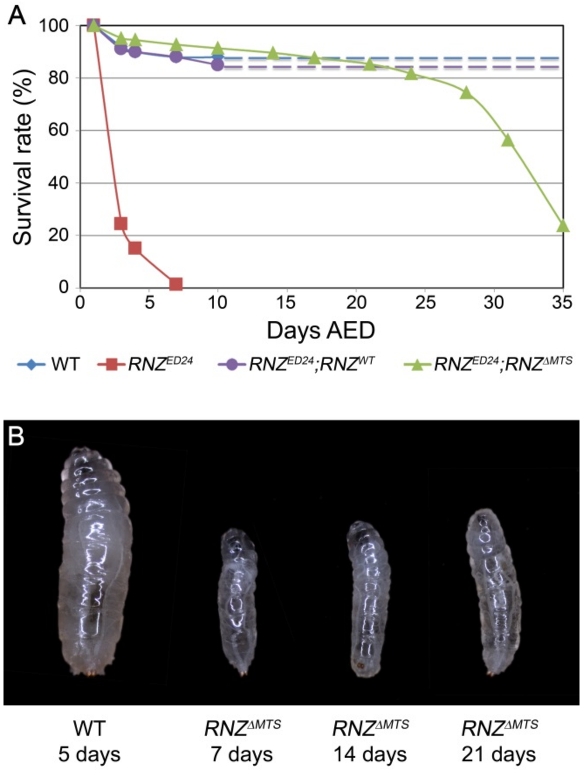

Figure S1. Mitochondrial dRNaseZ KO affects larval growth and survival. (*A*) Viability of WT (blue), RNZ^{ED24} KO (red), and $RNZ^{\Delta MTS}$ (green) or RNZ^{WT} (purple) rescued KO larvae animals was calculated as the variance from the initial number of 1st instar larvae. Dashed line indicates adult stage. (*B*) Representative examples of WT and $RNZ^{\Delta MTS}$ larvae. Samples were collected on 5th, 7th, 14th, and 21st day AED as indicated.

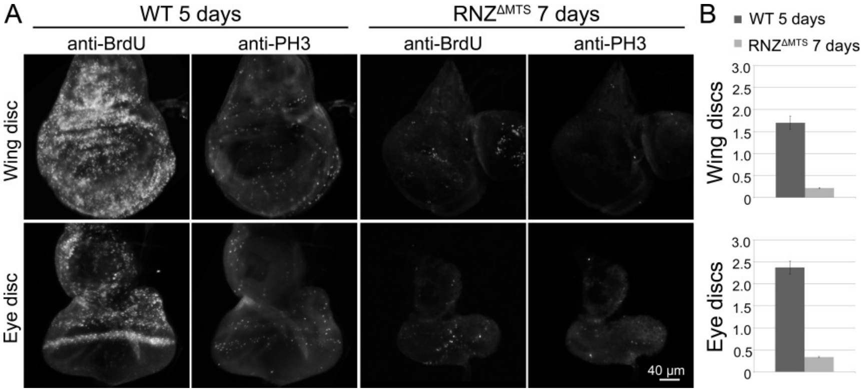

Figure S2. Mitochondrial dRNaseZ KO affects cell proliferation. (*A*) Third instar imaginal discs dissected from WT and *hsRNZ*-rescued $RNZ^{\Delta MTS}$ larvae. Discs were dissected on 5th and 7th day AED, and stained for BrdU and PH3. (*B*) Mitotic index of WT (dark grey) and $RNZ^{\Delta MTS}$ (light grey) imaginal discs. The mitotic index is calculated as the number of PH3-positive cells/area in μm×10^3.

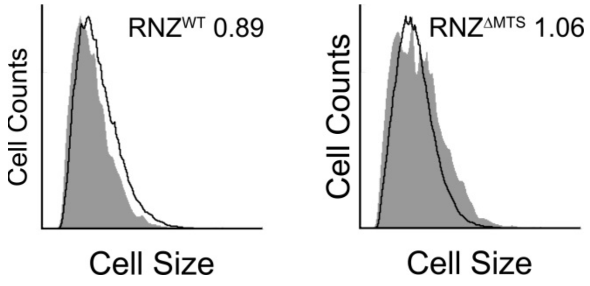

Figure S3. *RNZ*$^{\Delta MTS}$ cells have normal size. Forward scatter (FSC) analysis of wing discs containing *RNZ*^{WT} and *RNZ*$^{\Delta MTS}$ clones. Black traces correspond to the GFP-positive cells and gray filled traces correspond to GFP-negative. Numbers in the top right corner show the ratio of the mean sizes of GFP-negative cells (experimental population) versus GFP-positive cells (control population).

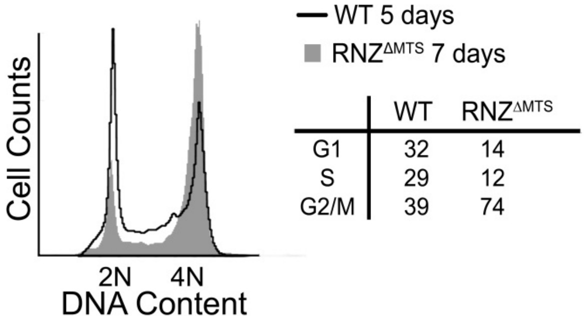

	WT	RNZ$^{\Delta MTS}$
G1	32	14
S	29	12
G2/M	39	74

Figure S4. *RNZ*$^{\Delta MTS}$ cells accumulate in G_2/M. Flow cytometry analysis of wing discs from WT and *hsRNZ*-rescued *RNZ*$^{\Delta MTS}$ larvae. Relative DNA content is shown on the X-axis, and cell count is shown on the Y-axis. Black traces correspond to the WT cells and gray filled traces correspond to the *RNZ*$^{\Delta MTS}$ cells. Table shows the percentage of cells in the G1, S and G_2/M phases in each group tested.

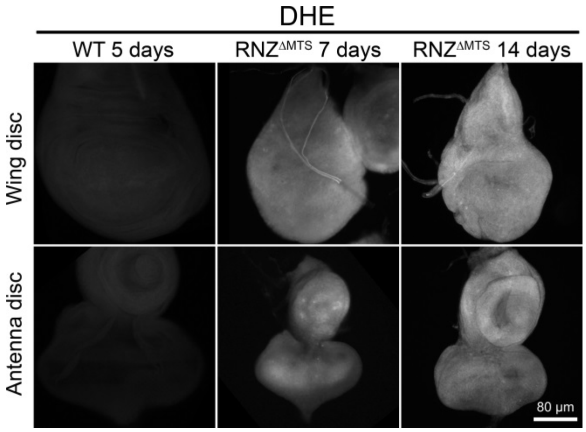

Figure S5. Mitochondrial dRNaseZ KO increases ROS production. Third instar imaginal discs dissected from WT and *hsRNZ*-rescued $RNZ^{\Delta MTS}$ larvae, and stained *in vivo* with DHE for the status of ROS.

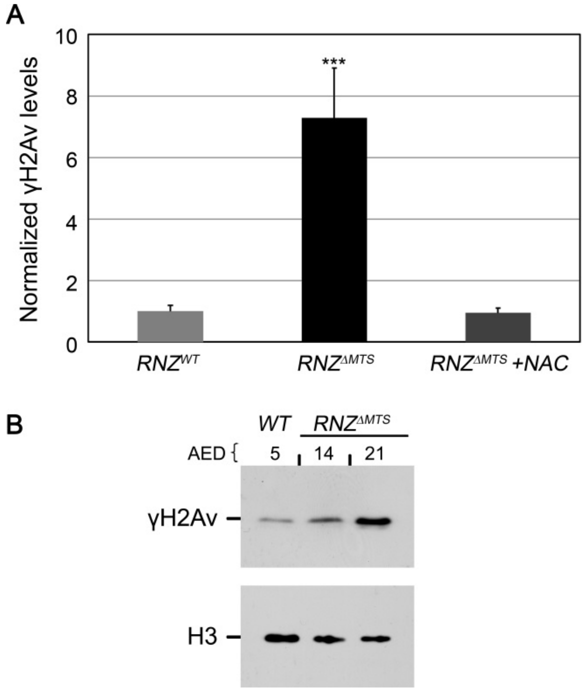

Figure S6. Mitochondrial dRNaseZ KO leads to ROS induced DNA damage. (*A*) Quantification of the number of γH2Av-positive cells per unit clone area. n=18 for each group. ***, p<0.001 from RNZ^{WT} clone. (*B*) Western blot analysis of γH2Av in wing discs dissected from WT and *hsRNZ*-rescued $RNZ^{\Delta MTS}$ larvae. Histone H3 (H3) is the loading control.

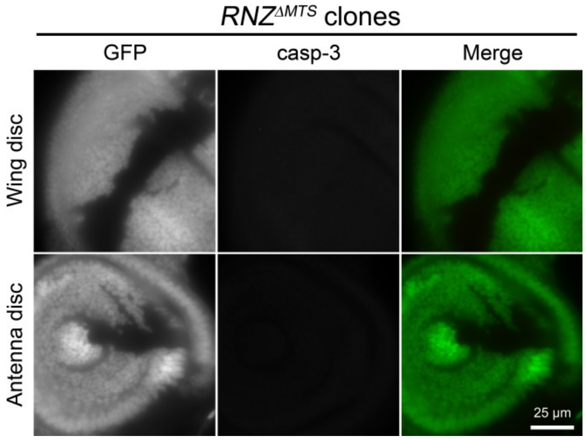

Figure S7. Mitochondrial dRNaseZ KO does not cause apoptosis. Imaginal discs with *RNZ*$^{\Delta MTS}$ clones generated on *Minute* background are stained using anti-cleaved caspase 3 antibody. Caspase activation is not detected.
